# Expansion Force‐Based Adaptive Multistage Constant Current Fast Charging with Lithium Plating Detection for Lithium‐Ion Batteries

**DOI:** 10.1002/advs.202504580

**Published:** 2025-05-19

**Authors:** Yudong Shen, Xueyuan Wang, Yuguang Li, Zhichao Zhang, Zhengde Tao, Yanan Hou, Xuezhe Wei, Haifeng Dai

**Affiliations:** ^1^ School of Automotive Studies Tongji University Shanghai 201804 China; ^2^ Tianmu Lake Institute of Advanced Energy Storage Technologies Co. Ltd. Liyang 213300 China

**Keywords:** charging rate self‐regulation, expansion force, fast charging, lithium‐ion battery, lithium plating detection

## Abstract

The multistage constant current (MCC) charging protocol for lithium‐ion batteries is commonly used to balance lithium plating and charging time. Traditional methods depend on a pre‐defined charging map without considering the feedback of lithium plating and subsequent self‐regulation of the charging rate. To tackle this problem, an adaptive MCC charging method is proposed, which is based on expansion force feedback to detect lithium plating. By integrating experiments with simulations, the results indicate that when lithium plating occurs, the force experiences an abnormal, accelerated increase. If the charging rate is reduced until lithium plating ceases, the force decreases. Correspondingly, three thresholds, V1, V2, and V3, in the derivative of force (dF/dSOC), are identified. Utilizing these thresholds, the charging rate can be self‐regulated. The results demonstrate that charging speed can be increased by 50% without causing irreversible lithium plating. The proposed method holds great promise for integration into intelligent battery management systems, thereby enhancing the performance of MCC fast charging.

## Graphical abstract

This article presents an adaptive multistage constant current (MCC) charging method for lithium‐ion batteries, using expansion force feedback to detect lithium plating in real time. It outlines how force‐derivative thresholds enable self‐regulation of the charging rate, increasing speed by 50% without damage. The approach promises significant improvements in intelligent battery management systems.

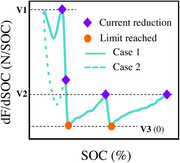

## 1 Introduction

Currently, lithium‐ion batteries (LIBs), which utilize graphite as the anode, are widely adopted as the energy source for electric vehicles (EVs).^[^ ^1^, ^2^ ^]^ Due to the alignment with the global trend of sustainable development, EVs have received significant policy support.^[^ ^3^, ^4^ ^]^ However, the market acceptance of EVs has not met expectations. A key reason is that, compared to the few minutes to refuel a conventional vehicle, the charging time for EVs typically takes tens of minutes or even hours, leading to range anxiety. As a result, improving the charging speed of EVs is an urgent issue that needs to be addressed.^[^ ^5^, ^6^ ^]^ The fundamental approach to reducing charging time is to increase the charging power of LIBs. For example, the U.S. Department of Energy has set a target for extreme fast charging at up to 400 kW.^[^ ^7^ ^]^ However, existing commercial LIBs struggle to maintain their lifespan and safety under such high charging power, as parasitic reactions are likely to occur.^[^ ^8^, ^9^ ^]^ Therefore, how to minimize charging time while maintaining LIB performance without degradation has attracted considerable attention.

Lithium (Li) plating, which directly compromises battery safety, accelerates capacity degradation and reduces lifespan, is the primary failure mode of LIBs under fast charging.^[^ ^10^, ^11^ ^]^ It refers to the phenomenon where Li ions fail to properly intercalate into the graphite anode during charging and instead deposit as Li metal on the graphite surface.^[^ ^12^ ^]^ Theoretically, Li plating is triggered when the anode potential drops too low. For graphite, the operating potential range is as low as 65–200 mV versus Li/Li^+^.^[^ ^13^, ^14^ ^]^ When the battery is charged at high current or high charging rates (C‐rate, the reciprocal of the time required to fully charge the battery), it causes significant polarizations, large charge‐transfer overpotentials, and/or sluggish kinetics.^[^ ^15^ ^]^ This lowers the anode potential below 0 V versus Li/Li^+^, leading to the occurrence of Li plating. It can also be triggered when charging under low temperature or high state of charge (SOC) conditions.^[^ ^16^ ^]^ Battery structure design,^[^ ^17^ ^]^ material composition,^[^ ^18^ ^]^ and enhancement treatments^[^ ^19^ ^]^ can also impact the fast charging capability of the battery. Li plating leads to the growth of Li dendrites, which can potentially puncture the separator, causing an internal short circuit in the LIBs, and in more severe cases, triggering fires and explosions.^[^ ^20^ ^]^ Furthermore, the deposited Li metal consumes the electrolyte and generates excessive solid electrolyte interphase (SEI). Additionally, some of the deposited Li metal may lose its electrochemical connection with the anode, resulting in the formation of dead Li. Both SEI and dead Li significantly reduce the available capacity of the LIBs.^[^ ^21^ ^]^ Therefore, the key challenge is to increase the charging rate while preventing Li plating. In practical fast charging, due to the lack of effective online detection methods for Li plating, conservative charge rates are typically used. This prevents the battery from fully realizing its charging potential, thus requiring longer charging time.

Various methods, such as acoustic techniques,^[^ ^22^, ^23^ ^]^ NMR,^[^ ^24^ ^]^ and implanted reference electrodes,^[^ ^25^ ^]^ can be used to detect Li plating. However, they require specialized lab equipment. While voltage relaxation^[^ ^26^ ^]^ and incremental capacity^[^ ^27^ ^]^ offer precision, they cannot detect the onset of Li plating and face robustness issues. Recently, methods that monitor the changing trends of battery displacement, force, or impedance to detect Li plating have attracted significant attention. These force‐based or impedance‐based methods are sensitive to Li plating and suitable for integration into battery management systems (BMS).^[^ ^28^, ^29^ ^]^ For instance, when Li plating occurs, the deposited Li metal causes a significant abnormal increase in thickness.^[^ ^29^, ^30^, ^31^ ^]^ The occurrence of Li plating also affects the charge transfer process, leading to an abnormal accelerated decrease in charge transfer resistance (*R*_ct_).^[^ ^32^, ^33^, ^34^ ^]^ However, the above methods mainly focus on Li plating detection without considering how to maximize the charging speed under controlled Li plating conditions. In practical fast charging, multi‐stage constant current (MCC) charging protocols are often used to suppress Li plating while reducing charging time.^[^ ^35^, ^36^, ^37^ ^]^ Therefore, a Li plating detection method under MCC charging needs to be proposed. Besides, after Li plating is detected, to adjust the charging rate needs to be adjusted. Furthermore, there is a lack of corresponding mechanistic models to assist in the study of the Li plating process.

To address the research gap, in this article, the variation of battery expansion force is investigated under MCC charging through experiments combined with simulations. Additionally, a Li plating detection criterion is proposed. Subsequently, an adaptive MCC fast charging strategy is implemented. Specifically, experiments are conducted to verify the effects of varying current, temperature, and charging rates on the battery expansion force. The origin of the battery expansion force during MCC charging is explained, detailing the variation of the expansion force during Li plating and the impact of the changed current on it. A thermo‐electro‐mechanical coupled model is also developed. Based on the expansion force feedback, an adaptive MCC fast charging method is implemented and further validated through long‐cycle tests. New Li plating detection standard and an optimized charging strategy are proposed, which can enhance fast charging performance. The main contributions of this paper are as follows:

The experimental results demonstrate the variation pattern of expansion force under MCC charging. Based on the differential expansion force (dF/dSOC), Li plating can be detected. A model that explains the mechanism of force variation and enables semi‐quantitative prediction of expansion force is established.

A new threshold (V2) based on dF/dSOC for Li plating detection is introduced, which greatly enhances detection sensitivity. Additionally, a target threshold (V3) for varying MCC current is proposed to prevent irreversible Li plating while boosting the charging speed.

By evaluating whether dF/dSOC meets the conditions of the V1, V2, and V3 thresholds, online Li plating detection and adaptive MCC fast charging are achieved. Experimental results show a 50% increase in charging speed without causing irreversible Li plating.

The remainder is structured as follows. The next section presents the experimental results and model simulation. Section 3 gives the adaptive MCC charging protocol. Section 4 provides the conclusion. The final section introduces the experiments.

## 2 Experimental Results and Model Simulation

In this section, the variation of expansion force during MCC charging is discussed in detail. Then, a detection method for Li plating under MCC is provided. Additionally, the changes in force during MCC charging are simulated through a model to better understand the role of force in Li plating. By combining experimental results with model simulations, this section lays the foundation for an online MCC current self‐regulating fast charging strategy based on force feedback.

### 2.1 Evolution of Mechanical, Electrical, and Thermal States

#### 2.1.1 One‐Stage MCC Charging

**Figure 1:**
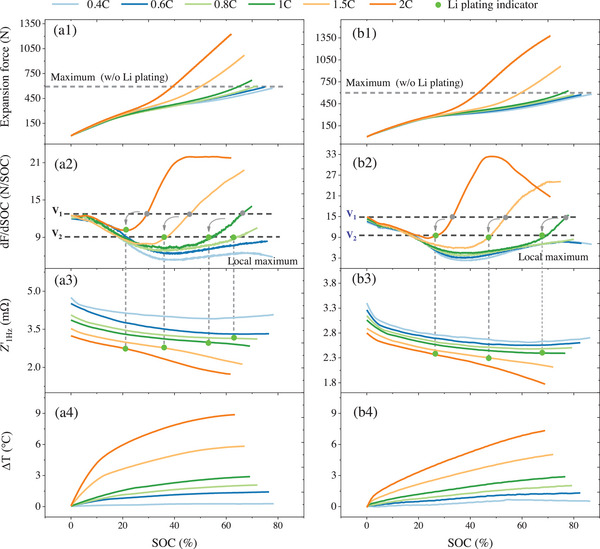
Experimental results of one‐stage MCC charging at 10 °C and 20 (C. a1–b4) are the results of expansion force, dF/dSOC, *Z'*_1Hz_, and △T, respectively.

The results under one‐stage MCC charging are presented, as shown in **Figure** **1**. The experiments were conducted at ambient temperatures of 10 and 20 °C, with charging rates ranging from 0.4 C to 2 C. The selection of 10 and 20 °C is due to these being commonly used temperatures for fast charging. For low‐temperature fast charging, the battery needs to be heated to ≈10 °C. A temperature of 20 °C meets the fast charging requirements. The results for other temperatures are consistent with these two temperatures. They are not shown here due to space limitations. Regarding the force variation, as shown in Figure 1a1,a2, it can be observed that, at low charging rates, the force increase trend for different rates is nearly identical, and the maximum expansion force eventually reached is also quite consistent. Furthermore, comparing the results at 10 ℃ and 20 °C, it can be concluded that the force variation pattern is almost independent of temperature. This is because, at low charging rates, the expansion force of the battery primarily results from the volume change of the active particles, which is only related to SOC. As the charging rate continues to increase, taking the results at 10 °C as an example, when the rate reaches 0.8 C, the expansion force exceeds the maximum expansion force observed at lower charging rates (≈590 N). As the charging rate increases further, the expansion force significantly exceeds 590 N, reaching ≈1230 N at 2 C. This significant increase in expansion force is caused by Li plating, which is confirmed.^[^ ^29^ ^]^

From a mechanistic perspective, during the charging process, the active material particles of the graphite anode and NCM cathode undergo different volume changes during the Li‐ion intercalation and deintercalation processes. This discrepancy results in an overall increase in the battery's thickness, leading to noticeable changes in the expansion force. Given the limited migration of Li‐ions during charging, there is an upper limit to the battery's expansion force, as shown by the maximum values in Figure 1a1,b1. When Li plating occurs, the volume change of the deposited Li metal is much larger than that of the anode and cathode materials, leading to a sharp increase in the expansion force, which can be clearly observed during the plating process.

Furthermore, the Li plating onset can be determined by the dF/dSOC curve, as shown in Figure 1b1. For low charging rates, the dF/dSOC curve initially decreases, reaching a minimum at ≈35% SOC, and then starts to rise again until the end of charging. It is noteworthy that the initial values of the dF/dSOC curve for different charging rates are essentially the same. This value is referred to as V1 in this article. In previous studies, the V1 has typically been used as an indicator threshold for Li plating detection.^[^ ^29^ ^]^ Although using V1 as an indicator of Li plating is generally effective, the value of V1 itself is relatively large, meaning that Li plating has already occurred. The real part of the impedance at 1 Hz (*Z'*_1Hz_) was also used to characterize Li plating.^[^ ^28^, ^38^, ^39^ ^]^ The inflection of the accelerated drop of the *Z'*_1Hz_ curve indicates the occurrence of Li plating, as shown in Figure 1a3.^[^ ^28^, ^38^, ^39^ ^]^ Based on the *Z'*_1Hz_ method, a new Li plating detection threshold, V2, was determined. Although V2 was established with the help of *Z'*_1Hz_, it can be found that at low charging rates, a local maximum occurs at the end of the charging process. This value is very close to V2. This result was also confirmed under different temperatures, as shown in Figure 1b2,b3. This characteristic provides great convenience for determining V2 under different operating conditions. V2 can be calibrated by completing a single charge at a low charging rate.

The statistical results of Li plating onset obtained from V1 and V2 are given in **Table** **1**. It can be seen that compared to V1, V2 can detect Li plating at least 5.7% SOC earlier.

**Table 1:** Statistical results of V1 and V2 under one‐stage MCC charging.

Temperature	Charging rate	V1 (SOC)	V2 (SOC)	Improved
10 °C	0.8 C	/	62.8%	/
	1 C	66%	53.5%	12.5%
	1.5 C	45.7%	40%	5.7%
	2 C	29.5%	21.3%	8.2%
20 °C	1 C	76.8%	68.4%	8.4%
	1.5 C	53.4%	47.2%	6.2%
	2 C	33%	26.5%	6.5%

The variation in the dF/dSOC curve is primarily due to the differing volume change rates of the graphite anode and NCM cathode. Taking the graphite anode as an example, during Li‐ion insertion, Li‐graphite compounds are formed. The rate of volume change varies with the degree of lithiation, which leads to changes in the slope of the dF/dSOC curve. V1 and V2 represent the transition points at the phase change locations. This change is intrinsic to the material's properties and will not be affected by changes in experimental conditions.

Although temperature has a significant effect on the occurrence of Li plating, the variation in temperature does not affect the expansion force change pattern under Li plating conditions. The high charging rate and/or low temperature can cause a significant force increase. Subsequent experimental and simulation results show that the thermal expansion displacement and thermal expansion force of the battery are much smaller compared to the intercalation expansion and Li plating expansion. Li plating can still be detected using V2. Since the effects of thermal expansion can be calculated and excluded, it is a significant advantage for the force‐based method to deal with large‐capacity batteries.

It is worth noting the significant additional expansion force increase that occurs during Li plating, which can potentially affect the battery's lifespan, capacity, and fast charging performance.^[^ ^40^ ^]^ Battery aging also leads to an increase in the battery's expansion force. However, this additional force needs to be considerably larger in order to have a noticeable impact on the battery's performance.^[^ ^41^ ^]^ This conclusion was confirmed in subsequent aging cycle experiments. Correspondingly, V1 and V2 are hence not significantly affected.

Based on the one‐stage MCC charging results, a new Li plating detection threshold, V2, for the force‐based method is established. The method for determining V2 is also provided. It is worth noting that when selecting different cathode and anode materials for the battery, the expansion force curve should be re‐evaluated to accurately determine the location of V2. Additionally, considering the delay in Li plating onset detection, further adjustments to the V2 value can be made in practical applications to reduce the risk of irreversible Li plating.

#### 2.1.2 Two‐Stage MCC Charging

**Figure 2:**
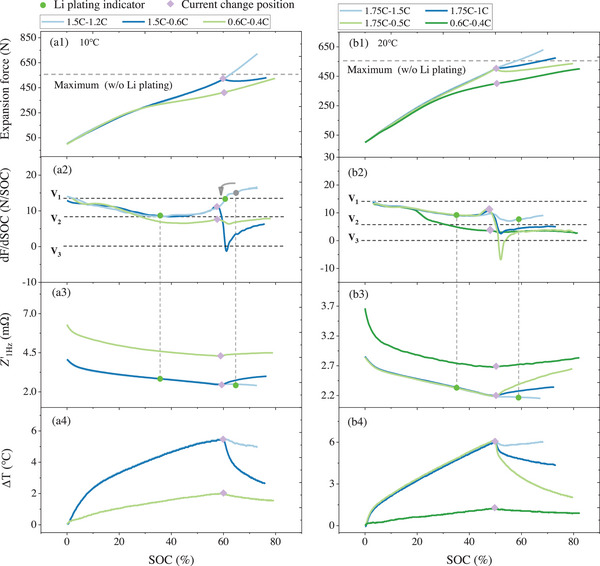
Experimental results of two‐stage MCC charging at 10 C and 20 C. (a1–b4) represents the results of the expansion force, dF/dSOC, *Z*'_1Hz_, and △T, respectively.

For two‐stage MCC charging, the charging rate is generally reduced for the subsequent charging process.^[^ ^42^, ^43^ ^]^ When the two charging stages are insufficient to cause Li plating, such as with 0.6 C–0.4 C charging, the maximum expansion force, as shown in **Figure** **2**a1,b1 for both 10 °C and 20 °C, does not exceed the maximum expansion force. Furthermore, the local maximum at the end of the dF/dSOC curve is set as V2, for the Li plating detection threshold. The V2 determined by this method is consistent with the results obtained by the *Z'*_1Hz_‐based method.

Li plating can also be determined through V2 for two‐stage MCC charging. When the charging rate is high enough to induce Li plating, the dF/dSOC remains consistently > V2, such as in the case of 1.5 C–1.2 C at 10 °C. Additionally, the local minimum value in the corresponding dF/dSOC curve can be considered as the Li plating onset indicator, which is also verified by the *Z'*_1Hz_‐based method. It is important to note that after reducing the charging rate, there is a period of rise for *Z'*_1Hz_, making it impossible to detect Li plating during this period. By comparison, the force‐based method still retains the ability to detect Li plating, despite the change of charging rate. The dF/dSOC curve does not show a decrease after the charging rate is reduced, indicating that the reduced rate still leads to Li plating. This is an advantage of the force‐based method.

Reducing the charging rate after the occurrence of Li plating, the dF/dSOC curve shows distinctive changes, depending on the charging rate applied. As shown in Figure 2b2, at 20 °C, the charging rate is maintained at 1.75 C until 50% SOC. The expansion force increases rapidly. Depending on V2, it can be determined that Li plating occurred at ≈35% SOC. When the charging rate is reduced to 1.5 C, the value of dF/dSOC remains above V2, which indicates the Li plating has not disappeared. Cross‐validation with the *Z'*_1Hz_‐based method confirms that Li plating still occurs. When the charging rate drops to 1 C, the value of dF/dSOC remains consistently below V2, indicating that the rate of 1 C cannot cause Li plating. When the current rate drops to 0.5 C, as shown in Figure 2b1, a significant decrease in expansion force can be observed. This originates from the re‐intercalation of plated Li. The corresponding dF/dSOC value quickly falls below zero. This phenomenon points out the changing target for the charging rate after Li plating, which is to decrease the charging rate until the dF/dSOC value drops below zero. The value of dF/dSOC at zero is defined as V3. The value of V3 is confirmed to result from the expansion force drop induced by Li reinsertion. While changes in experimental conditions may affect its process, they will not influence the force drop phenomenon.

The statistical results V1, V2, and V3 are given in **Table** **2**. Under varying current conditions, the charging rate in the second stage must be reduced to a sufficiently low level to meet the V3 threshold.

**Table 2:** The results of V1, V2, and V3 under two‐stage MCC charging.

Temperature	Charging rate	V1 (SOC)	V2 (SOC)	Improved	V3 (SOC)
10 °C	1.5 C–1.2 C	/	35.9%	/	/
	1.5 C–0.6 C	/	35.9%	/	60.7%
20 °C	1.75 C–1.5 C	/	35%	/	/
	1.75 C–1 C	/	35%	/	/
	1.75 C–0.5 C	/	35%	/	51.2%

By defining V2 and V3, the method for detecting Li plating and adjusting the charging rate is established. When dF/dSOC remains/rises above V2, it can be determined that Li plating occurs. After Li plating, the charging rate must be reduced until dF/dSOC falls below V3 to ensure that no Li plating would occur again. It is important to note that when Li plating is detected through V2, it occurs during the Li nucleation stage. At this point, the Li plating is reversible.^[^ ^44^ ^]^

#### 2.1.3 Three‐Stage MCC Charging

**Figure 3:**
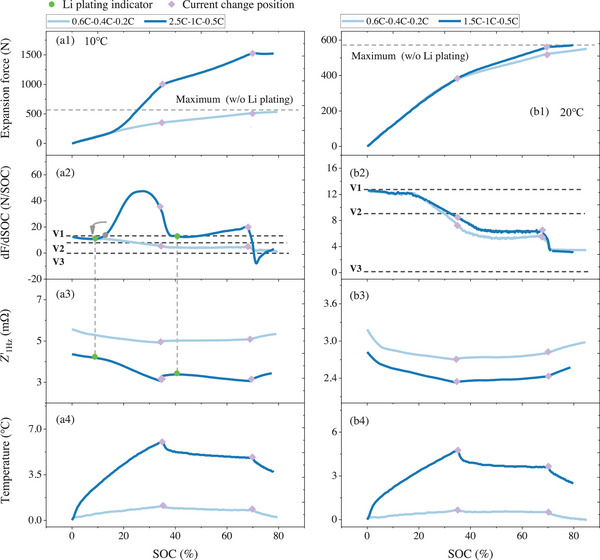
Experimental results of three‐stage MCC charging at 10 ℃ and 20 ℃. (a1–b4) refers to the results of the expansion force, dF/dSOC, *Z*'_1Hz_, and △T, respectively.

For a three‐stage MCC charging, the conclusions mentioned above remain consistent. As shown in **Figure** **3**a1, for the case of 2.5 C–1 C–0.5 C at 10 °C, the first charging stage (2.5 C) causes severe Li plating, leading to a rapid increase in battery expansion force.

The corresponding dF/dSOC curve, shown in Figure 3a2, allows for a quick determination of Li plating depending on V2. Even when the charging rate is reduced to 1C, the expansion force does not decrease, and the corresponding dF/dSOC remains above V2, indicating that the rate reduction is insufficient to stop Li plating. As the current is further reduced to 0.5 C, the expansion force begins to decrease, and the corresponding dF/dSOC quickly falls below V3, indicating that 0.5 C is not enough to sustain Li plating. When applying low charging rates, such as 0.6 C–0.4 C–0.2 C, both the expansion force and dF/dSOC meet the condition to avoid Li plating. Even with a higher rate in the initial stage, as shown in Figure 3b1, when charging at 1.5 C, as long as the rate is reduced to 1 C and 0.5 C before Li plating occurs, Li plating can be prevented. Note that the combination of 1.5 C–1 C–0.5 C MCC charging rate at 20 °C is significantly higher than the recommended charging rate. By this force feedback‐based approach, the fast charging potential of the battery can be effectively utilized.

The statistical results V1, V2, and V3 are shown in **Table** **3**. Based on the results, the charging rate affects the trend of the dF/dSOC curve. Therefore, it is important to flexibly choose between V1 or V2 for diagnosing Li plating, rather than relying solely on one, as V1 and V2 may not satisfy the conditions simultaneously. Using either of them, depending on the situation, can effectively determine Li plating.

**Table 3:** The results of V1, V2, and V3 under the three‐stage MCC charging.

Temperature	Charging rate	V1 (SOC)	V2 (SOC)	Improved	V3 (SOC)
10 ℃	2.5 C–1 C–0.5 C	13%–40.8%	9%–/	4%–/	/–70.4%

### 2.2 Development and Validation of Model

#### 2.2.1 Thermo‐Electro‐Mechanical Model Development

**Figure 4:**
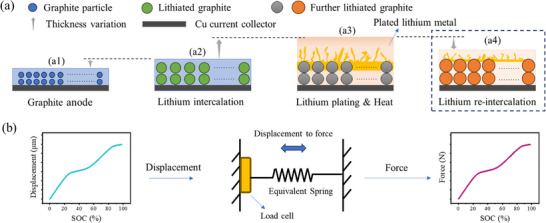
Battery expansion mechanism and calculation method. a) The expansion of the battery is caused by graphite lithiation, Li plating, and heat generation. b) Calculation of expansion force.

As shown in **Figure** **4**a1,a2, during the charging process, the battery thickness increases continuously with the lithiation of graphite.^[^ ^45^ ^]^ This is because the volume change of graphite particles is ≈13%, while it is only ≈3% for NCM.^[^ ^44^ ^]^ For the graphite‐NCM material system used in this experiment, the change in battery thickness is primarily governed by the graphite anode. When Li plating occurs, as shown in Figure 4a3, a significant increase in battery thickness can be observed.^[^ ^29^ ^]^ When the charging rate is reduced to a level where Li plating no longer happens, the thickness experiences obviously decrease, as shown in Figure 4a4. This is because the plated Li re‐intercalates into the graphite anode, which is confirmed by the experiments mentioned above. The battery is typically a volume‐limited system, and thus, the increase in thickness is eventually translated into the expansion force.

A thermo‐electro‐mechanical coupled model for the battery is established. This model simulates the variation in behavior of the expansion force under MCC charging and explains the underlying mechanism of these changes. Furthermore, the model provides a semi‐quantitative prediction of the expansion force variation under Li plating conditions, which can be used to optimize the adaptive MCC charging process.

The electrochemical model is developed based on the P2D model, considering the processes of Li plating and Li re‐intercalation. The basic equations of the P2D model include the conservation of mass and charge in both the solid and liquid phases, as well as the electrochemical reaction kinetics equations that describe the electrochemical reaction rates at the electrode/electrolyte interface. Detailed equations and boundary conditions can be found in the reference.^[^ ^46^ ^]^ The Li plating and Li re‐intercalation reactions in the battery can be described by the Butler‐Volmer equations, as shown in Equations (1 and 2).

(1) $$j_{pl}=i_{0,pl}\;\left[\exp\left(\frac{\alpha_{a,pl}F}{RT}\eta_{pl}\right)-\exp\left(-\frac{\alpha_{c,pl}F}{RT}\eta_{pl}\right)\right],\;\eta_{pl}<0$$

(2) $$j_{re}=\varepsilon_{re}i_{0,re}\left[\exp\left(\frac{\alpha_{a,re}F}{RT}\eta_{re}\right)-\exp\left(-\frac{\alpha_{c,re}F}{RT}\eta_{re}\right)\right]$$

where, *j* is the current density, while *i*_0_ is the exchange current density. The parameters *α_a_* and *α_c_* are the transfer coefficients for the anode and cathode, respectively. *F* is the Faraday constant. The overpotential is denoted as *η*. *R* is the gas constant, and *T* is the temperature. The subscripts *pl* and *re* represent the Li plating and Li re‐intercalation processes, respectively. *ε*_re_ represents the percentage of reversible Li.

The intercalation of Li‐ions induces diffusion‐induced stress, causing strain in the radial direction and leading to the expansion of the active particles. The relevant processes are represented by Equations ((3), (4), (5)).^[^ ^47^ ^]^ The radial expansion of the active particles at a radius of *m* can be described by Equation (3).

(3) $$\begin{array}{ccc} u(m) & = & m[\frac{2\Omega (1-2v)}{9(1-v)}\frac{3}{M^{3}}\int_{0}^{M}c_{s}\left(m\right)m^{2}dm\\ & & +\frac{\Omega (1+v)}{9(1-v)}\frac{3}{m^{3}}\int_{0}^{m}c_{s}\left(m\right)m^{2}dm] \end{array}$$

where *Ω*, *ν*, *c_s_*, *m*, and *M* represent the material's partial molar volume, Poisson ratio, solid‐phase Li concentration, particle radius, and maximum particle radius, respectively.

The maximum expansion (*u*_max_) of the active particles occurs at the surface. Setting *m* = *M* in Equation (3) and treating the active particles as spheres, Equation (3) can be approximately calculated by Equation (4). Where, *c_s,avg_* is the average solid‐phase lithium concentration.

(4) $$u_{\max}=\frac{\Omega M}{3}\;c_{s,avg}$$

Considering the change in the thickness direction, the intercalation expansion △*L_int_* of the battery can be expressed by Equation (5).

(5) $$\Delta \;L_{int}=\sum \left(\varepsilon_{l,n}\frac{3u_{\max,n}}{M_{n}}L_{0,n}\right)$$

where *ε_l_* and *L*_0_ represent the particle‐electrode expansion conversion coefficient and the initial thickness of the electrode, respectively, with the subscript *n* denoting the number of electrode layers.

The thermal expansion △*L_q_* is given by Equation (6), where, *α_bat_* is the battery's thermal expansion coefficient, *L_bat_* is the original thickness of the battery, and *T_0_* is the ambient temperature. The heat generation in the battery is modeled by a 1D thermal model. The detailed equations and boundary conditions can be found in the reference.^[^ ^48^, ^49^, ^50^ ^]^ The electrochemical model and the thermal model of the battery are coupled in both directions.

(6) $$\Delta L_{q}=\alpha_{bat}\int_{0}^{L_{bat}}\left(T-T_{0}\right)dL$$

The effect of Li plating on the thickness can be expressed by Equation (7).^[^ ^51^ ^]^ Equation (7) illustrates that the volume change caused by Li plating is much larger than that of intercalation. Compared to the other, it is more than four times larger. Notably, the model developed in this study is used for the semi‐quantitative prediction of Li plating to study the changing trend of expansion force. The related effects of Li plating, such as SEI film thickening and gas generation, are not modeled. The uneven distribution of Li plating on the anode surface has also been considered to further improve the modeling accuracy.

(7) $$V\left(Q_{pl}\right)=\frac{Q_{pl}}{eN_{a}}\;\left(V_{m,pl}-6\alpha_{\mathrm{Li}C_{6}}V_{m,gr}\right)$$

Where, *V_m, pl_* and *V_m, gr_* represent the molar volume of Li and graphite, respectively. *Q_pl_* refers to the amount of plated Li. *N_a_* is the Avogadro constant, and *e* is the elementary charge. *α*_LiC6_ is the coefficient of expansion changing from C to LiC_6_.

The total thickness (or the displacement) change (△*L*) of the battery during the charging process can be expressed by Equation (8). It consists of three components: the intercalation expansion △*L_int_*, the thermal expansion △*L_q_*, and the Li plating expansion △*L_pl_*.

(8) $$\Delta L=\Delta L_{int}+\Delta L_{q}+\Delta L_{pl}$$

△*L* is typically very small (measured in µm), which is not suitable for online applications. A load cell is commonly used to amplify the expansion displacement and convert it into expansion force. Figure 4b illustrates the method for converting expansion displacement to expansion force. The battery can be equivalently modeled as a spring. Hence, its total expansion force △*F* is given by Equation (9), where *k_e_* is the equivalent stiffness coefficient of the battery. The equivalent stiffness coefficient of the battery was calibrated using a charging rate of 0.2 C. The expansion, displacement, and expansion pressure of the battery were measured as a function of SOC. Subsequently, the equivalent stiffness coefficient of the battery was calculated based on a unified SOC scale.^[^ ^52^, ^53^ ^]^ Equation 9 illustrates the established conversion relationship between displacement and force.

(9) $$\Delta F=k_{e}\cdot \Delta L$$

The model mentioned above is constructed in COMSOL ver5.5. The model parameters are given in Table S1 (Supporting Information). The model parameters are derived from measurements or literature, and some of them are fitted using the experimental results.

#### 2.2.2 Model Validation

**Figure 5:**
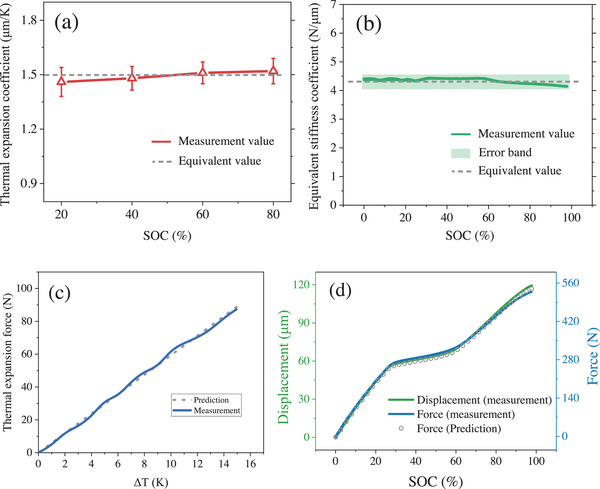
Measurement and prediction results. a) Thermal expansion coefficient at different SOC. b) Battery equivalent stiffness coefficient. c) Thermal expansion force. d) Displacement and force under quasi‐steady state.

The results of the thermal expansion coefficient are shown in **Figure** **5**a. The thermal expansion coefficient changes slightly with SOC. For convenience, a uniform value of 1.5 µm K^−1^ is used. The equivalent stiffness coefficient of the battery is shown in Figure 5b. Considering the limited variation in the equivalent stiffness coefficient, which ranges from 4.18 to 4.39 N µm^−1^, the equivalent stiffness coefficient is uniformly taken as 4.28 N µm^−1^ to simplify the calculation of expansion force. The approximated values for the thermal expansion coefficient and equivalent stiffness coefficient are reasonable, as a semi‐quantitative estimate is sufficient to meet the needs of the study. After obtaining the thermal expansion coefficient and equivalent stiffness coefficient of the battery, the expansion force can be calculated based on the expansion displacement, which is given by Equation (9).

The result of the thermal expansion force is shown in Figure 5c. It can be observed that the predicted value is very close to the measured value, demonstrating its effectiveness. During the experiment, the maximum temperature rise of the battery did not exceed 9 °C, resulting in a thermal expansion force of <50 N, which is much lower than the force induced by Li plating. The result of the expansion force originating from the intercalation of Li‐ions is shown in Figure 5d. It can be seen that the trends in displacement and force are almost identical, indicating a nearly linear relationship between displacement and force. The result shows that the difference between the experimental and predicted values of expansion force is very small.

**Figure 6:**
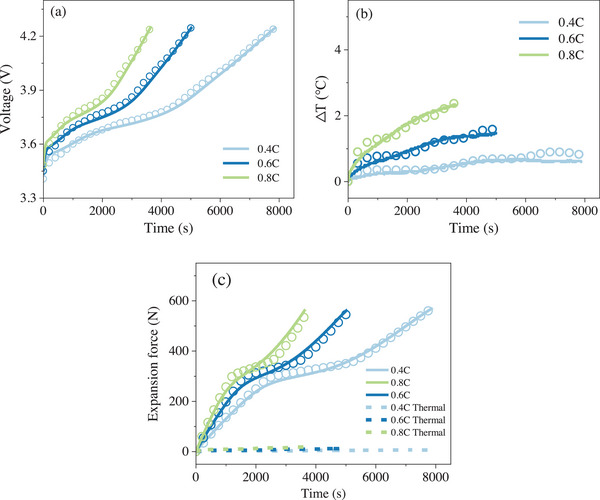
Comparison of the model and experimental results. (a–c) presents the results of voltage, temperature difference, and expansion force, respectively.

The comparison between the model results and experimental results for different charging rates under non‐Li plating conditions at 20 °C is shown in **Figure** **6**. The model‐predicted voltage and temperature fit the experimental results well. Additionally, the predicted battery expansion force also aligns closely with the experimental data. The root mean square errors (RMSE) for voltage, temperature, and expansion force are no >19.9 (mV), 0.15 (°C), and 23.8 (N), respectively. Compared to the intercalation expansion force, the thermal expansion force can be neglected, as displayed in Figure 6c. These results indicate that the established model has sufficient accuracy.

**Figure 7:**
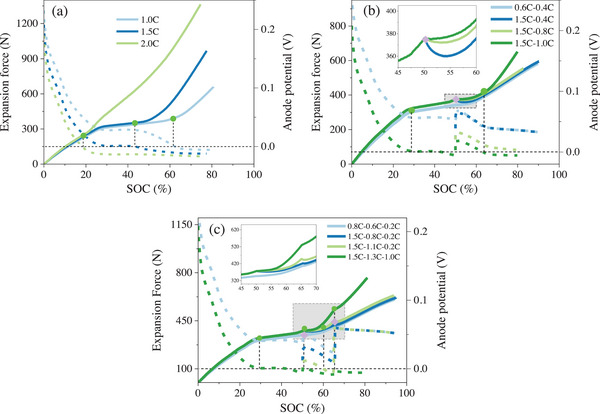
Simulation results of the model for expansion force and anode potential. (a–c) refers to the results of one‐stage, two‐stage, and three‐stage MCC charging, respectively.

By the effective prediction of the battery's expansion force, the simulation of the expansion force under different operating conditions was further completed by the developed model. Based on the model, the anode potential can be directly obtained to determine the onset of Li plating, and the changes in expansion force can be simultaneously studied. Li plating is considered to occur when the anode potential falls below 0 V versus Li/Li^+^. The model simulated the changes in force and anode potential under Li plating conditions for one‐ to three‐stage MCC, and the results are shown in **Figure** **7**. Under the one‐stage MCC charging condition, as the charging rate increases from 1 C to 2 C, the Li plating onset shifts from 62% SOC to 18% SOC. The corresponding positions are marked on the expansion force curve, as indicated by the green dots. It can be observed that after Li plating occurs, the increase in expansion force starts to accelerate. As the charging rate increases, both the slope and the maximum of the expansion force curve significantly increase. This trend aligns with the experimental results shown in Figure 1. When the charging stages are increased to two stages, the change in battery expansion force under different Li plating conditions shows a significant difference. Under non‐Li plating conditions (0.6 C–0.4 C), the battery expansion force increases steadily, primarily due to the volume change of active particles during charging. Using a 1.5 C charging rate to induce Li plating, and reducing it to 0.8 C and 0.4 C to prevent further Li plating, which leads to a significant decrease in the expansion force. This is because the plated Li re‐intercalates into the anode. Notably, when the charging rate is reduced to 1C, the anode potential briefly rises above 0 V versus Li/Li^+^. After 15% SOC, Li plating resumes. The corresponding force curve shows that the expansion force does not decrease but starts to increase at a slower rate. The results of the dF/dSOC curve obtained from the simulation are similar to those derived from the experiments. This trend is consistent with the experimental results. Increasing the charging stages to three, as shown in Figure 7c, leads to similar conclusions.

The results obtained from the model simulation further validate the conclusions drawn from the experiments. Under MCC charging, Li plating can be determined by the expansion force and the corresponding dF/dSOC curve. When the force or the dF/dSOC exceeds a given threshold, it is determined that Li plating has occurred. After reducing the charging rate, if the force decreases or dF/dSOC falls below 0, it can be determined that the reduced charging rate no longer causes Li plating. After detecting Li plating, quickly reducing the charging rate does not cause irreversible Li plating because the Li plating is in the lithium nucleation stage.^[^ ^44^ ^]^

## 3 Adaptive MCC Charging Protocol

Developing a fast charging protocol for batteries is often very challenging because it requires considering many factors, such as temperature, SOC, and aging, to balance charging time with the likelihood of Li plating. In practice, the inconsistency of battery operating conditions is even higher. This inconsistency is not only evident within different batteries in the same battery pack but also between batteries in different vehicles. It is difficult to use a fixed charging protocol to address the significant differences between batteries. This leads to the occurrence of Li plating and faster degradation of the battery, even though the charging protocol has been carefully designed. The root of the problem lies in the fact that the charging protocol is open‐loop, meaning it cannot adjust the charging rate based on the battery's actual Li plating status. Therefore, it is necessary to adjust the charging rate in real time to an appropriate level based on the actual Li plating state. This requires a closed‐loop adaptive MCC charging method with Li plating feedback.

### 3.1 Principle and Implementation

**Figure 8:**
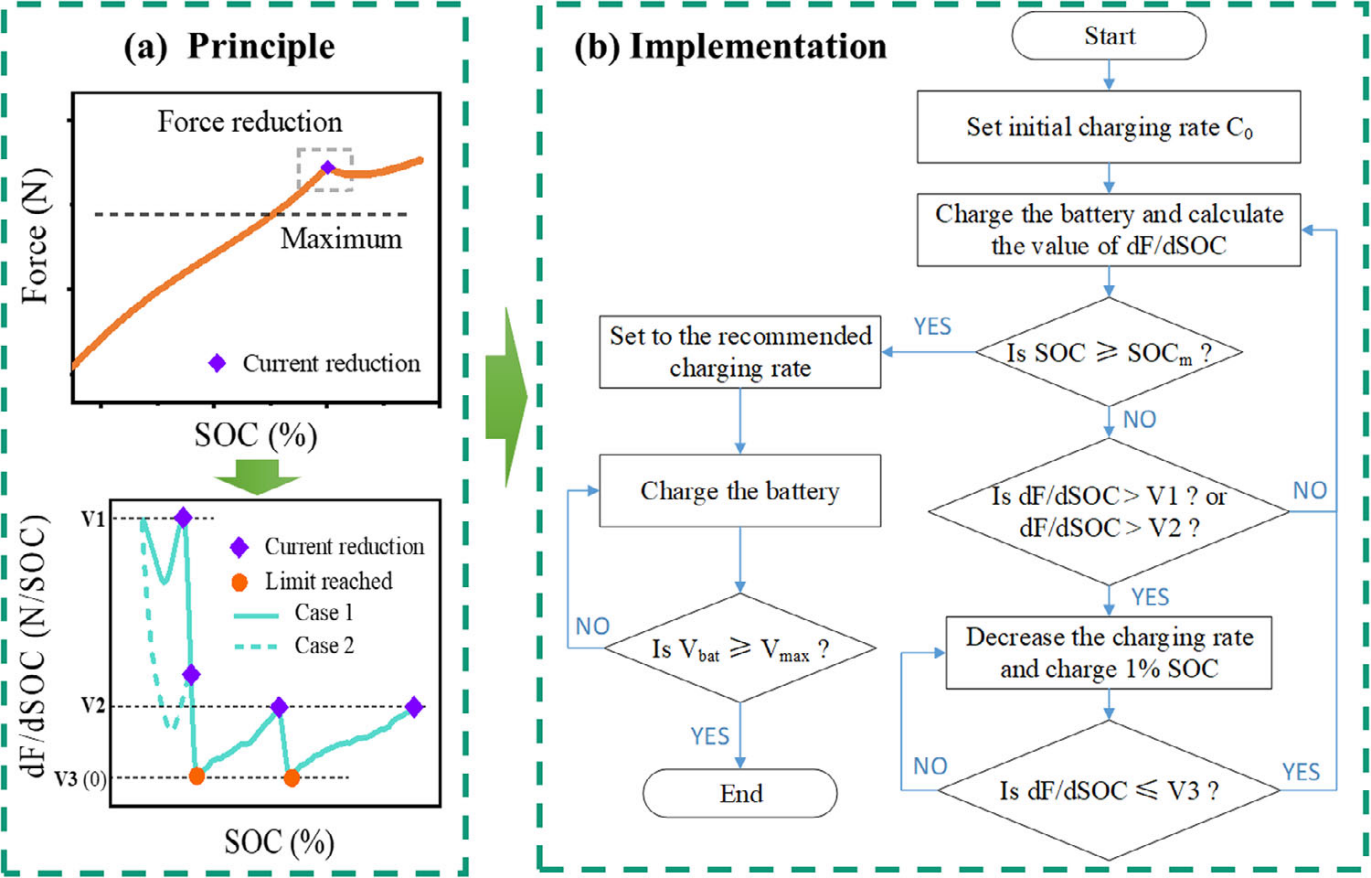
Diagram of force feedback‐based closed‐loop adaptive MCC charging. a) Principle. b) Implementation.

According to experiments and simulations, as shown in **Figure** **8**a, it is confirmed that during MCC charging, Li plating causes a significant acceleration in the increase of expansion force, which far exceeds the normal maximum expansion force. Besides, when the charging rate is reduced to a level where Li plating no longer happens, the expansion force obviously decrease, which can be attributed to the re‐intercalation of plated Li. Based on the dF/dSOC, the state of Li plating can be detected, and a closed‐loop fast charging protocol can be implemented, which increases the charging speed without causing irreversible Li plating. As shown in Figure 8a, when it is found that dF/dSOC exceeds V1 (case 1) or V2 (case 2), Li plating is determined to have occurred. The charging rate should be reduced until dF/dSOC decreases to V3. Then, the charging continues at this rate. If dF/dSOC rises and reaches V2 again, the above process is repeated.

A force‐feedback‐based adaptive MCC fast charging strategy is proposed, as shown in Figure 8b. As a concept validation, a host computer program simulating BMS functionality was developed. Based on the actual experimental data, the charging strategy is formulated. It is important to note that during fast charging, the safety and lifespan of the battery should be prioritized. This means that the charging strategy should be conservative, rather than focusing solely on minimizing charging time. The charging process is carried out according to the predefined charging steps. Specifically, a high charging rate that is much higher than the recommended charging rate is used as the initial charging rate (C_0_) for the battery. When dF/dSOC exceeds V1 or V2, it is determined that Li plating has occurred. At this point, the charging rate must be reduced to prevent irreversible Li plating. In this experiment, the charging rate is reduced in a step of 0.25C, and dF/dSOC is checked every 1% SOC until it is less than or equal to V3. Afterward, the charging continues with the reduced charging rate until the charging process is completed. It is important to monitor whether the current SOC has reached the preset SOC_m_ during charging. The purpose of setting SOC_m_ is to ensure that the battery can be fully charged and the plated Li can re‐intercalate into the anode as much as possible, preventing irreversible Li plating. Once this condition is met, the recommended charging rate should be applied until the battery voltage (V_bat_) reaches the upper cutoff voltage (V_max_).

### 3.2 Validation

**Figure 9:**
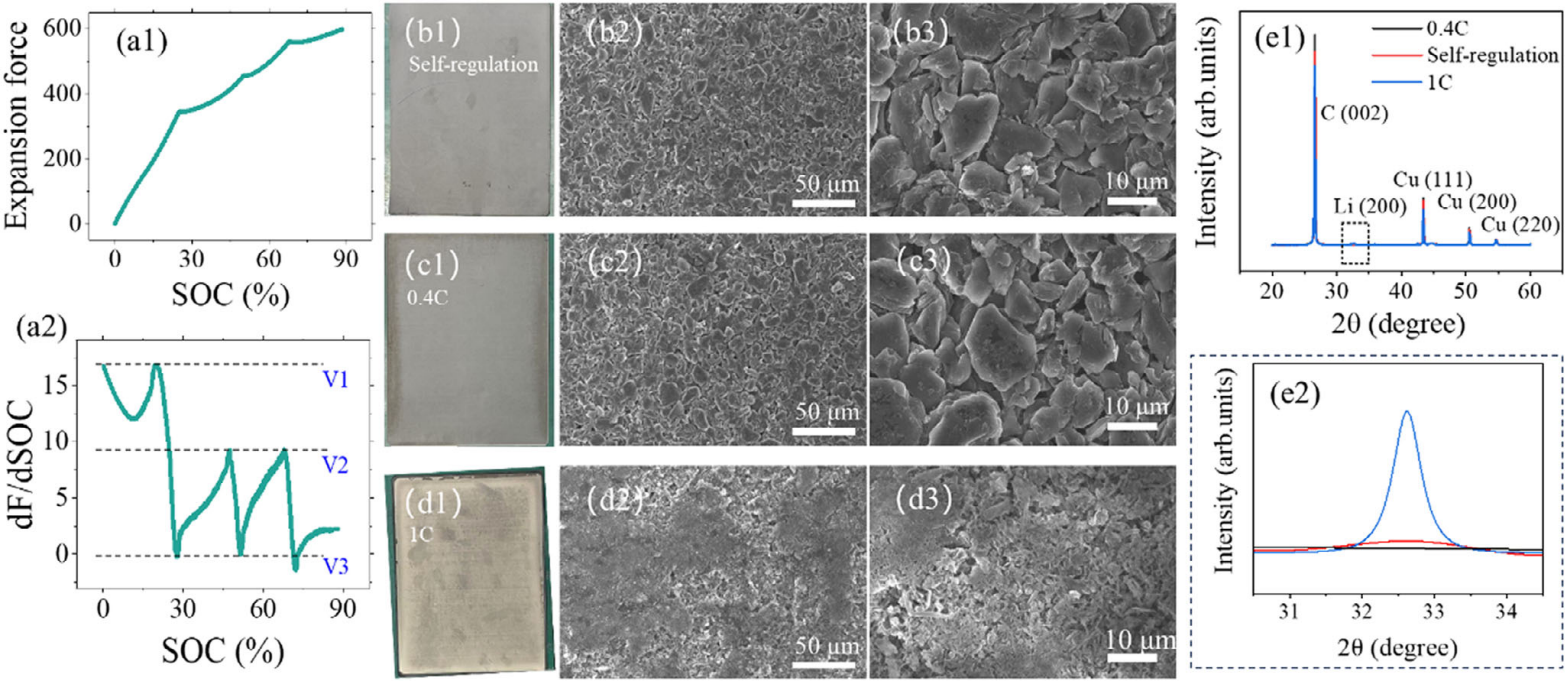
Experimental results under 100 cycles. (a1) is the expansion force variation in the last cycle, and (a2) is the corresponding dF/dSOC curve. (b1–d3) are the optical photos and SEM results. (e1) is the XRD result, and (e2) is the corresponding local magnification.

This charging process was repeated 100 times. The average charging rate during the process was higher than 0.6C. The expansion force and dF/dSOC curves for the last cycle are shown in **Figure** **9**a1,a2. Additionally, 0.4 C and 1 C charging rates were added as controls. The 0.4 C rate is slightly higher than the recommended charging rate, while 1 C significantly exceeds the recommended rate.

After the aging cycles, optical photos, SEM, and XRD were used to characterize the surface of the graphite anode. The results of optical photos and SEM are shown in Figure 9b1–d3. It can be seen that in the cases of self‐regulation and 0.4 C, the optical photos show a grayish‐black surface on the electrode. Besides, the SEM images show clean graphite particles with no debris. It indicates that no Li plating occurred on the graphite surface.^[^ ^41^, ^54^ ^]^ In contrast, with 1 C charging, the optical photo shows a silvery‐white surface, and the SEM image reveals that the graphite surface is covered with mossy Li and Li dendrites, making the graphite particles unrecognizable. This indicates irreversible Li plating.^[^ ^55^ ^]^ The XRD results further support this conclusion. As shown in Figure 9e1,e2, a characteristic peak of metallic Li appears under 1 C charging, while the other two do not.

The results of the cycling aging indicate that the self‐regulating charging method can avoid irreversible Li plating while increasing the charging speed by 50%.

## 4 Conclusion

In this article, an online Li plating detection method based on expansion force during the MCC charging process is proposed. Additionally, MCC fast charging with charging rate self‐regulation is realized through force feedback. The main conclusions are summarized as follows:

During MCC charging, the expansion increases rapidly, induced by Li plating. When the charging rate is insufficient to sustain Li plating, the force decreases. The decrease in expansion force is due to the re‐intercalation of plated Li. Both experimental and simulation results jointly validate this conclusion.

New thresholds (V2 and V3) of dF/dSOC are proposed for Li plating detection and charging rate self‐regulation during MCC charging. Compared to V1, V2 has higher sensitivity in Li plating detection. After Li plating, the charging rate should be reduced until dF/dSOC reaches threshold V3 to avoid irreversible Li plating. Methods for determining thresholds V2 and V3 are provided.

By comparing dF/dSOC with the given thresholds V1, V2, and V3, the state of Li plating can be determined online, and the charging rate can be adjusted purposefully. Long‐cycle results show that this method ensures no irreversible Li plating occurs while increasing the charging speed by 50%.

In future work, we plan to further investigate different types of materials for electrodes (such as LFP) to determine the applicability of the force‐based method. Besides, we will conduct more research on the relationship between expansion force changes and Li plating in battery modules.

## 5 Experimental Section

### Experimental Objects

All experiments were conducted on pouch batteries with a nominal capacity of 33.3 Ah. Detailed information is provided in **Table** **4**.

**Table 4:** Specifications of the experimental battery.

Item	Value
Manufacturer	Farasis Energy
Model	IMP06160231P32B
Capacity	33.3 Ah (25 ℃)
Size	231×160×6.05 (mm, L×W×H)
Cathode materials	NCM
Anode materials	Graphite
Cathode/anode layers	17 / 18
Operational voltage	3 V to 4.25 V
Standard charging rate	0.33 C at 25 ℃ a
Maximum charging rate	1 C at 25 ℃

The battery samples were selected based on several key criteria. First, batteries with relatively large capacities were chosen to facilitate the measurement of expansion forces. Additionally, the significant heat generation during the fast charging process makes these batteries suitable for studying thermal expansion behaviors. The NCM/Graphite electrode material combination, which is one of the most commonly used in commercial batteries, was also selected. It is important to note that the batteries used in this study are commercially available. These selection criteria ensure that the findings of this research are generalizable and applicable to real‐world scenarios.

### Experimental Setup

**Figure 10:**
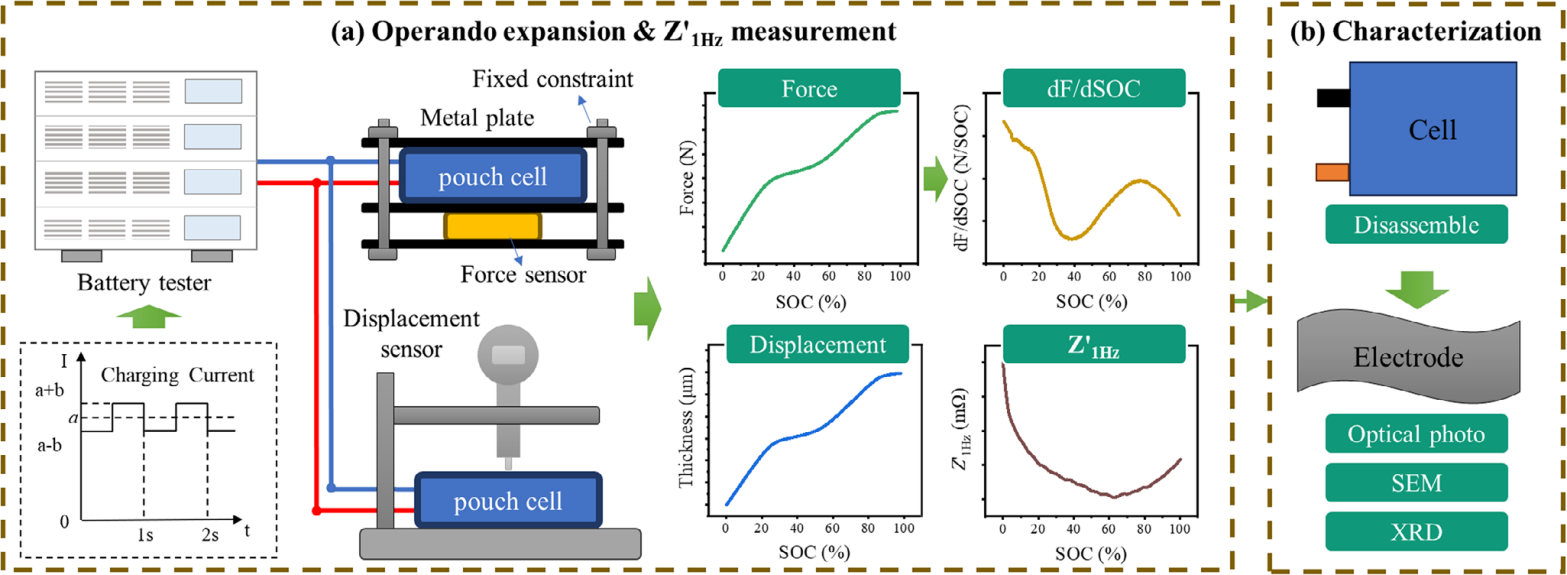
Illustration diagram. a) The setup of the operando measurement system and the acquisition of expansion force and dF/dSOC, expansion displacement, and *Z'*_1Hz_ curves at the cell level. b) Cell characterization.

The charge‐discharge experiments of the tested batteries were conducted using a battery testing system (Stropower, CTSH‐6‐120‐16ISO). Voltage and current data during the charge‐discharge process were synchronously collected through a host computer connected to the testing system. The battery was placed in a chamber to maintain environmental temperature stability. Battery temperature was measured using a T‐type thermocouple. The thermocouple was placed at the positive tab of the battery. An illustration diagram is displayed in **Figure** **10**.

As shown in Figure 10a, the expansion of the battery was measured in two parts: displacement and force. The displacement of the battery, corresponding to its free expansion, was measured. A displacement sensor (Xiwak, 1127‐01) with a precision of 0.1 µm was used for this measurement. Figure 10a shows the basic setup of force measurement. A metal block, slightly larger than the battery, was attached to the battery as a force‐distribution plate. The expansion force was measured using a load cell (Rullide, RDF‐610) with a precision of 1N, which was attached to the metal block. The entire setup was then clamped into a bench.

The *Z'*_1Hz_ measurement was directly conducted using the battery testing system. Specifically, a small‐amplitude square wave current with a period of 1s was superimposed on the constant charging current, and the composite current was injected into the battery as the excitation. The superimposed charging current waveform, resembling a pulse current, is shown in Figure 10a. Here, *a* represents the target charging current magnitude, and *b* denotes the superimposed amplitude. The final magnitudes are *a*+*b* and *a*‐*b*, respectively. The response voltage of the battery was synchronously collected, and *Z'*_1Hz_ was calculated through a fast Fourier transform (FFT) based on the excitation current and response voltage. More details can be found in the previous work.^[^ ^28^, ^38^ ^]^

After the experiments, the batteries were further characterized, as shown in Figure 10b. The batteries were disassembled in a glove box filled with argon gas. The disassembled anode was cleaned three times with dimethyl carbonate (DMC). The surface of the anode was characterized using SEM (HITACHI 8100) and XRD (Bruker D8 ADVANCE).

### Experimental Procedures

The experimental ambient temperatures were set to 10 ℃ and 20 °C. The batteries were tested using one‐stage MCC, two‐stage MCC, and three‐stage MCC charging protocols, with charging rates ranging from 0.2 C to 2 C. Charging was stopped once the battery reached the upper cutoff voltage without a constant voltage (CV) charging process. The results of more stages of MCC charging experiments can be extrapolated from the results of the one‐ to three‐stage MCC charging. After charging, the temperature was changed to 25 °C, and the battery was discharged at a rate of 0.5 C to the lower cutoff voltage. In all the above experiments, the batteries were loaded into the device shown in Figure 10a and subjected to an initial preload force of 700N, which is the recommended value. Before the experiments, all tested batteries were cycled five times at a low rate using a constant current constant voltage (CCCV) charging protocol at 25 °C to maintain chemical stability. Data from the final cycle were used to calibrate the battery capacity.

The coefficient of thermal expansion of the battery was calibrated using an alternating current heating method, as described in.^[^ ^56^ ^]^ The temperature rise and thermal expansion were measured at SOCs of 20%, 40%, 60%, and 80%. Then, the thermal expansion coefficient of the battery can be calculated.

## Conflict of Interest

The authors declare no conflict of interest.

## Supporting information



Supporting Information

## Data Availability

The data that support the findings of this study are available from the corresponding author upon reasonable request.
