# Supplementary material for: Expansion Force‐Based Adaptive Multistage Constant Current Fast Charging with Lithium Plating Detection for Lithium‐Ion Batteries
Source: Adv Sci (Weinh). 2025 May 19;12(30):e04580. doi: 10.1002/advs.202504580 (PMC12376661; doi:10.1002/advs.202504580)
Supplement: Supplementary file 1 — Supporting Information [file ADVS-12-e04580-s001.docx]

**Expansion Force-based Adaptive Multistage Constant Current Fast Charging with Lithium Plating Detection for Lithium-ion Batteries**

*Yudong Shen ^a^, Xueyuan Wang ^a,^*, Yuguang Li ^a^, Zhichao Zhang ^b^,* *Zhengde Tao ^b^,* *Yanan Hou ^b^, XueZhe Wei ^a^, Haifeng Dai ^a,^***^[[1]](#footnote-1)^

**^a^** School of Automotive Studies, Tongji University, Shanghai 201804, China

**^b^** Tianmu Lake Institute of Advanced Energy Storage Technologies Co., Ltd., Liyang 213300, China

**Table S1**. Parameters used in the thermo-electro-mechanical model.

| **Symbol** | **Description** | **Unit** | **Value** | | |
| --- | --- | --- | --- | --- | --- |
| **Constant** | | | | | |
| *F* | Faraday constant | C·mol^-1^ | 96485.3383 |  |  |
| *R* | Ideal gas constant | J·mol^-1^·K^-1^ | 8.314 |  |  |
| *Brug* | Bruggemann coefficient | 1 | 1.5 |  |  |
| *T*_ref_ | Reference temperature | K | 298.15 |  |  |
| $N_{a}$ | Avogadro constant | mol^-1^ | 6.022×10^23^ |  |  |
| **Geometric properties** | | | **Anode** | **Separator** | **Cathode** |
| $L_{0}$ | Length | m | 1×10^-4 a^ | 7×10^-5 a^ | 3×10^-5 a^ |
| *M* | Particle radius | m | 2×10^-6 a^ | / | 2×10^-6 a^ |
| *ε*_s_ | Volume fraction of the solid phase | 1 | 0.6 ^a^ | 0.5 ^a^ | 0.6 ^a^ |
| *ε*_e_ | Volume fraction of the liquid phase | 1 | 0.4 ^a^ | 0.5 ^a^ | 0.34 ^a^ |
| *A*_s_ | Specific surface area | m^-1^ | 3*ε*_s_/*R*_p_ | / | 3*ε*_s_/*R*_p_ |
| **Transport properties** | | | **Anode** | **Separator** | **Cathode** |
| *D*_s_ | Solid phase diffusion coefficient | m^2^·s^-1^ | 1.45×10^-13 c^ | / | 5×10^-13 c^ |
| *D*_e,ref_ | Liquid phase diffusion coefficient at *T*_ref_ | m^2^·s^-1^ | / | $10^{-9.4339+0.0002c_{e}-2\times10^{-7}c_{e}^{2}}$ ^b^ | / |
| $D_{e}^{\mathrm{eff}}$ | Effective liquid phase diffusion coefficient | m^2^·s^-1^ | $\varepsilon_{s}^{\mathrm{Brug}}$*D*_e,ref_ | $\varepsilon_{s}^{\mathrm{Brug}}$*D*_e,ref_ | $\varepsilon_{s}^{\mathrm{Brug}}$*D*_e,ref_ |
| *σ*_s_ | Solid phase electrical conductivity | S·m^-1^ | 100 ^b^ | / | 10 ^b^ |
| $\sigma_{s}^{\mathrm{eff}}$ | Effective solid phase electrical conductivity | S·m^-1^ | $\varepsilon_{s}^{\mathrm{Brug}}\sigma_{s}$ | / | $\varepsilon_{s}^{\mathrm{Brug}}\sigma_{s}$ |
| *σ*_e,ref_ | Liquid phase electrical conductivity at *T*_ref_ | S·m^-1^ | / | $5\times10^{-10}c_{e}^{3}-2\times10^{-6}c_{e}^{2}+0.0025c_{e}$ ^b^ | / |
| $\sigma_{e}^{\mathrm{eff}}$ | Effective liquid phase electrical conductivity | S·m^-1^ | $\varepsilon_{e}^{\mathrm{Brug}}\sigma_{e}$ | $\varepsilon_{e}^{\mathrm{Brug}}\sigma_{e}$ | $\varepsilon_{e}^{\mathrm{Brug}}\sigma_{e}$ |
| *c*_e,0_ | Initial value of Li^+^ concentration in the liquid phase | mol·m^-3^ | / | 1000 ^b^ | / |
| *c*_s,max_ | Maximum value of Li^+^ concentration in the solid phase | mol·m^-3^ | 31507 ^c^ | / | 50060 ^c^ |
| *c*_s,0_ | Initial lithium concentration of electrode | mol·m^-3^ | 1360 ^c^ | / | 46656 ^c^ |
| *t*_+_ | Transference number | 1 | / | 0.363 ^b^ | / |
| **Kinetics properties** | | | **Anode** | **Separator** | **Cathode** |
| α_a,1_, α_c,1_ | Transfer coefficients of lithium intercalation reaction | 1 | 0.5, 0.5 ^b[1]^ | / | 0.5, 0.5 ^b[1]^ |
| *k* | Reaction Rate Constant | m/s | 7e-11 ^c^ | / | 5e-11 ^c^ |
| *U*_ref_ | Open circuit potential at *T*_ref_ | V | Fig. S1 (a) ^a^ |  | Fig. S1 (b) ^a^ |
| $\alpha_{a,pl}$, $\alpha_{c,pl}$ | Transfer coefficients of lithium plating reactions | 1 | 0.3, 0.7 ^b[1]^ | / | / |
| $\alpha_{a,re}$, $\alpha_{c,re}$ | Transfer coefficients of lithium re-intercalation reactions | 1 | 0.3, 0.7 ^b[1]^ |  |  |
| $i_{0,pl, ref}$ | Reference exchange current density of lithium plating and stripping reactions | A·m^-2^ | 1300 ^c^ | / | / |
| *U*_Li_ | Equilibrium potential of lithium plating and stripping reactions | V | 0 ^b^ |  |  |
| *M*_Li_ | Molar mass of lithium | kg·mol^-1^ | 0.00694 ^b^ | / | / |
| *ρ*_Li_ | Density of lithium | kg·m^-3^ | 534 ^b^ | / | / |
|  | | | | | |
| **Thermal properties** | |  | **Anode** | **Separator** | **Cathode** |
| *ρ*_cell_ | Density of the battery | kg·m^-3^ | 2300 ^b^ | 900 ^b^ | 4870 ^b^ |
| *C*_p_ | Thermal capacity | J·kg^-1^·K^-1^ | 1437.4 ^b^ | 2050 ^b^ | 1150 ^b^ |
| *h* | Thermal conductivity | W·m^-1^·K^-1^ | 1 ^b^ | 2.16 ^b^ | 1.58 ^b^ |
| dU/dT | entropy coefficient | V·K^-1^ | Fig. S2 (a) ^b[2]^ | / | Fig. S2 (b) ^b[2]^ |

1. ^a^The parameters that are derived from measurements or battery manufacturer.
2. ^b^The parameters that are derived from the cited literature or the comsol material database.
3. ^c^The parameters that are evaluated within practical ranges to fit the experimental data.

**References**

[1] P. Arora, M. Doyle, R. E. White, *J. Electrochem. Soc.* **1999**, *146*, 3543.

[2] I. Baghdadi, O. Briat, A. Eddahech, J. M. Vinassa, I. Baghdadi, P. Gyan, in *2015 IEEE 24TH INTERNATIONAL SYMPOSIUM ON INDUSTRIAL ELECTRONICS (ISIE)*, IEEE, New York **2015**, pp. 1248–1252.

1. *Corresponding author. Email: 7wangxueyuan@tongji.edu.cn

   **Corresponding author. Email: tongjidai@tongji.edu.cn [↑](#footnote-ref-1)
